# Selective deficits of S-cone in thyroid-associated ophthalmopathy patients without clinical signs of dysthyroid optic neuropathy

**DOI:** 10.3389/fnins.2022.990425

**Published:** 2022-09-21

**Authors:** Haochen Jin, Xi Yu, Suqi Cao, Mengting Wang, Xiaozhou Hu, Jie Ye, Weijie Liu, Mingna Xu, Wencan Wu, Yunhai Tu

**Affiliations:** State Key Laboratory of Ophthalmology, Optometry and Vision Science, School of Ophthalmology and Optometry, Affiliated Eye Hospital, Wenzhou Medical University, Wenzhou, China

**Keywords:** S-cone, color vision, visual impairments, thyroid-associated ophthalmopathy, dysthyroid optic neuropathy

## Abstract

**Purpose:**

We explored whether thyroid-associated ophthalmopathy (TAO) patients without clinical signs of dysthyroid optic neuropathy (DON) would have a selective deficit mediated by S-cone.

**Methods:**

Thirty-two TAO patients without clinical signs of DON (non-DON, 42.03 ± 9.59 years old) and 27 healthy controls (41.46 ± 6.72 years old) participated in this prospective, cross-sectional study. All observers were tested psychophysically after passing color screening tests and a comprehensive ocular examination. Isolated L-, M-, and S-cone contrast thresholds were measured at 0.5 cyc/deg using Gabor patches. We calculated the area under the receiver operating characteristic (ROC) curve to quantify the ability of chromatic contrast sensitivity to detect the early visual function changes in non-DON patients.

**Results:**

S-cone contrast sensitivity in non-DON patients was found to be lower than that of healthy controls (*P* < 0.001), whereas the sensitivities to L- and M-cone Gabor patches were similar between these two groups (*P* = 0.297, 0.666, respectively). Our analysis of the ROC curve revealed that the sensitivity to S-cone had the highest index to discriminate non-DON patients from healthy controls (AUC = 0.846, *P* < 0.001). The deficit of S-cone was significantly correlated with muscle index in non-DON patients (*R* = 0.576, *P* = 0.001).

**Conclusion:**

There is a selective S-cone deficit in the early stage of TAO. S-cone contrast sensitivity could serve as a sensitive measure of visual impairments associated with early DON in patients with TAO.

## Introduction

As a severe complication of thyroid-associated ophthalmopathy (TAO), dysthyroid optic neuropathy (DON) is one of the leading causes of permanent visual impairments, poor quality of life and reduced social interactions (Miskiewicz et al., 2016; Bruscolini et al., 2018). Early diagnosis is critical in treating DON. Without timely diagnosis and treatment, there is a risk of irreversible sight loss (Verity and Rose, 2013; Dolman, 2021). Currently, diagnosis of DON is made mainly based on decreased visual acuity (VA), visual field (VF) defects, relative afferent pupillary defect, and significant orbital apex crowding (Blandford et al., 2017). Most of these abnormalities occur in patients who show obvious optic nerve deficits. It is still a challenge to diagnose DON at its early stage before the aforementioned visual impairments are noticed (Mckeag et al., 2007).

On the other hand, color vision has been proven to be a sensitive diagnostic tool in optic neuropathy (Almog and Nemet, 2010). Trichromatic human color vision is mediated by the three types of retinal cone photoreceptors that are sensitive to long (L-cone), medium (M-cone), and short (S-cone) wavelengths of light (Sharpe et al., 1999). The absence or dysfunction of any type is potentially at risk for specific color vision deficits. Also, S-cones are unique as they are relatively sparse in the retina and are relatively vulnerable to damage (Hood et al., 1984; Greenstein et al., 1989). It is also clear that the S-cones comprise between 5 and 10% of all cones in the primate retina. Hence, even if there is a fixed proportion loss of the three types of cone photoreceptors, deficits of S-cone might be more easily revealed (Curcio et al., 1991; Wool et al., 2019). Several studies have found selective S-cone deficits in the early stage of optic nerve relevant diseases, even before the appearance of initial clinical signs. For example, Anssari et al. (2019) found a significant deficit of S-cone at 2 cyc/deg in both early and established multiple sclerosis patients without a history of optic neuritis. Ong et al. (2003) evaluated the effect of sight threatening diabetic retinopathy on color vision, and found that these patients had a significantly worse S-cone contrast sensitivity despite with normal VA. Moreover, several studies also reported that S-cone deficits could be present before peripheral VF loss in glaucoma (Drance et al., 1981; Adams et al., 1982; Bayer et al., 2020). These studies indicate that S-cone function might be a sensitive measure of early optic nerve dysfunction.

In our study, we investigated whether there would be a selective deficit mediated by S-cone in TAO patients without clinical signs of DON. We asked this question for two reasons. First, similar to the aforementioned optic nerve relevant diseases that affect S-cone sensitivity, DON could also be characterized by optic nerve deficits (Neigel et al., 1988). To illustrate, Zhang et al. (2019) found that changes of retinal microvasculature, as represented by the peripapillary capillary vessel density, could be associated with the severity of DON. Wu et al. (2020) reported the thickness of retinal nerve fiber layer and ganglion cell complex around macular significantly decreased in DON patients. Moreover, deficits in color vision are common in patients with DON (Neigel et al., 1988; Tanner et al., 1995; Mckeag et al., 2007; Wong et al., 2018; Garip-Kuebler et al., 2020; Garip-Kuebler et al., 2021). For example, Mckeag et al. (2007) reported that 77% of eyes with definite DON, 56% of eyes with equivocal DON, and even 7% eyes without DON experienced color vision deficits. Although changes of color vision in patients with DON have been well documented in previous studies, the type and severity of color vision deficits, especially in TAO patients without clinical signs of DON, are not clear. For this reason, we systematically evaluated L-, M-, and S-cone contrast sensitivity in TAO patients with normal VA, VF, optic nerve head appearance, and no signs of orbital apex crowding. We show that there is a selective S-cone deficit in TAO patients before the clinical signs of DON manifest.

## Materials and methods

### Observers

Thirty-two TAO patients without clinical signs of DON (non-DON group, mean age 42.03 ± 9.59 *SD*, 15 males, 17 females) and 27 age- and sex-matched healthy controls (control group, mean age 41.46 ± 6.72 *SD*, 12 males, 15 females) participated in our study. They were consecutively recruited between February 2021 and September 2021 in the Affiliated Eye Hospital of Wenzhou Medical University, Wenzhou, Zhejiang, China. All patients with TAO were diagnosed according to the Bartley international diagnostic criteria (Bartalena et al., 2008) by the last author. Exclusion criteria were those with congenital color vision deficits, refractive errors over +1.50 diopters (D) or under −4.00 D of spherical equivalent or 1.50 D of astigmatism, VA impairment (logMAR best corrected VA, > 0), VF changes with a mean deviation <−2 dB, optic disk edema, evident orbital apex crowding, corneal involvement, significant media opacities, any neurologic or systemic disease (rather than thyroid disorders), and previous diagnosis of glaucoma, uveitis, retinal, or optic nerve diseases. The clinical activity of TAO was graded according to the clinical activity score scheme (Bartalena et al., 2008), and patients with clinical activity score ≥ 3/7 were in the stage of inflammation. This study was performed in accordance with the tenets set forth in the Declaration of Helsinki and was approved by the ethics committee of the Eye Hospital of Wenzhou Medical University (2020-106-K-93-01). Written informed constant was obtained from each participant before the experiment.

### Clinical examinations

**FIGURE 1:**
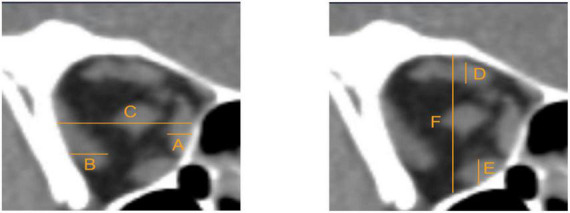
Measurements were taken at the coronal CT scan halfway between the posterior globe and orbital apex. **(A)** Medial rectus muscle. **(B)** Lateral rectus muscle. **(C)** Orbital width. **(D)** Superior rectus/levator complex. **(E)** Inferior rectus muscle. **(F)** Orbital height. The horizontal muscle index = (A+B)/C. The vertical muscle index = (D+E)/F. The larger one of these two indexes was taken as final muscle index.

All the TAO patients underwent a comprehensive ocular examination, including slit-lamp biomicroscopy, refraction and best corrected VA test, VF test using the 30–2 strategy (Humphrey Field Analyzer II; Carl Zeiss Meditec, Inc., Dublin, CA, USA), non-contact intraocular pressure measurement (Topcon, Tokyo, Japan), exophthalmometry measurements, ophthalmoscopy, and orbital CT scans. Based on the method described by Barrett et al. (1988), we used the muscle index on orbital CT scans as an index of orbital apex crowding. We conducted measurements at the coronal CT scan halfway between the posterior globe and orbital apex. Firstly, we obtained the transverse dimensions of the medial rectus muscle, lateral rectus muscle and orbital width along a horizontal line through the optic nerve. And then we calculated the horizontal muscle index as the percentage of orbital width occupied by the medial and lateral rectus muscles. Similarly, we measured the longitudinal dimensions of the superior rectus/levator complex, inferior rectus muscle and orbital height along a vertical line through the optic nerve. And then we calculated the vertical muscle index as the percentage of the orbital height occupied by the superior rectus/levator complex and inferior rectus muscle (Figure 1). The larger one of these two indexes was taken as the final muscle index. Ishihara pseudoisochromatic plates was performed to exclude participants with congenital color vision deficits. Twenty-eight of the patients completed the biological indicators of thyroid, such as free triiodothyronine and free thyroxine.

One eye of each subject was selected; If both eyes met the eligibility criteria of the study, the right eye was chosen to undergo the cone contrast test and then analyzed.

### Apparatus

Cone contrast thresholds of subjects were measured by Psykinematix v2.4.2 software (KyberVision, Sendai, Miyagi, Japan) run on an iMac (retina 4K, 21.5-inch, 2019) which had a mean measured luminance of 26 cd/m^2^. During the whole experiment, we conducted a color calibration using the Radiometric Mode in Psykinematix (Beaudot, 2009) and MCS-100 Spectroradiometer (Hangzhou SENSING Co., Ltd.),^1^ and used Spider5Elite colorimeter (Datacolor, Lawrenceville, New jersey, USA)^2^ to calibrate the luminance of the display every 2 months. Contrast thresholds were estimated using a two-down one-up staircase method with an accuracy of 79.1%. The stimulus contrast reduced at a rate of 50% after two correct responses which would change to 12.5% after the first reversal, whereas the contrast increased after one incorrect response and the increase rate was always 25%. The initial contrast values were, respectively: L-cone 10.39%, M-cone 10.39%, S-cone 91.80%. Each staircase was terminated after six reversals, and chromatic contrast thresholds were calculated as the mean of the last five reversals.

### Stimuli

**FIGURE 2:**
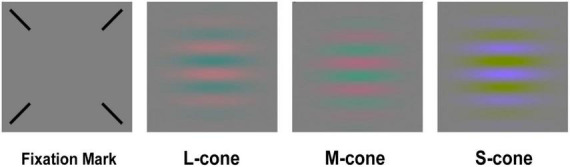
The fixation and L-, M-, and S-cone Gabors.

Our test stimuli were oriented Gabor patches (spatial frequency of 0.5 cyc/deg and a 2D Gaussian envelope with a sigma of 2 deg). The Gabor patches were in sine phase relative to the center of the screen, and their sinusoidal components were modulated along three cone-isolating directions (L, M, and S) (Figure 2). The cone-isolating stimuli were created through the use of “silent substitution” methods (Estévez and Spekreijse, 1982), which involved modulating the red, green, and blue guns of the iMac display to alter a single cone’s response: quantal catch rates in one cone class increased or decreased without affecting the quantal catch rates in the other two cone classes. It is widely used in the study of color vision psychophysics (Wang et al., 2014; Shepard et al., 2016; Taylor et al., 2018). The cone excitations of the L, M, and S cones were calculated using Smith and Pokorny’s cone fundamentals (Smith and Pokorny, 1975). The stimulus strength was defined in cone contrast units. For example, for the L-cone Gabor patch, cone contrast is:


$$\frac{\text{Δ}\,L}{L}=\frac{L\,p\,e\,a\,k-L\,b\,g}{L\,b\,g}$$

The Equation on the right refers to L-cone quantal catch, where L_*peak*_ is the maximum quantal catch produced by the Gabor, and L_*bg*_ is the background catch produced by a gray mean field. M- and S-cone contrasts were defined in the same way. A noise-bit technique (Allard and Faubert, 2008) was used to enable a high resolution.

### Procedure

The experiment was tested within a dark environment and the stimuli were presented on a mean gray background as shown in Figure 2. Every participant’s head was stabilized with a chin rest at a viewing distance of 52 cm. A fixation mark (four thin black diagonal lines, and each pointing at the center of the screen) was presented throughout the experiment. In each trial, subjects were asked to finish a two-interval forced choice (2-AFC) task: in the first place, a 333 ms interval with an auditory signal with the fixation mark in the absence (foil interval) or the presence of the test stimulus (target interval) was shown to subjects. Then, a 400 ms inter-stimulus blank interval with the fixation mark alone was shown. Subsequently, a second 333 ms interval with the auditory signal, fixation mark was presented in the absence or the presence of the test stimulus depending on whether the first interval was a foil or target interval. The order of target and foil interval was randomized, and subjects were asked to report the interval during which they perceived the test stimulus with a keyboard. After each trial, auditory feedback in response to correct and incorrect responses. Cone contrast sensitivity was monocularly measured with individual’s optimal spectacle correction, and the untested eye was covered with a patch. Before the test, subjects were given 5 min to adapt to the dark test environment, during which they were given practice trials to become familiar with the experimental setting and procedure.

### Statistical analysis

Data were presented as means ± standard deviation and statistical analysis was performed using statistical software (SPSS version 26; IBM Corp., Armonk, NY, USA). The spherical equivalent of refractive error was calculated as the spherical dioptric power plus one half of the cylindrical dioptric power. Independent samples *t*-test was used to compare the differences in means between the control group and non-DON group. The difference of sex between the two groups was assessed with the χ^2^ test. The receiver operating characteristic (ROC) curve was calculated to evaluate the ability of cone contrast sensitivity and to determine early visual impairments in the TAO patients without clinical signs of DON. Larger areas under the ROC curve (AUC) indicated higher diagnostic value. The correlations between the cone contrast sensitivity and clinical parameters were tested with Pearson’s or Spearman’s correlation analysis. A *p*-value less than 0.05 was deemed as statistically significant.

## Results

### Patient characteristics

The clinical characteristics of participants are provided in Table 1. There were no significant differences in age, sex distribution, spherical equivalent, intraocular pressure between the non-DON and control groups (Ps > 0.17). In terms of visual functions, the non-DON and control groups did not differ significantly in their best corrected VA, mean deviation or pattern standard deviation of VF (Ps > 0.48). In the non-DON group, the mean exophthalmometry was 19.03 ± 2.44 mm (range: 13–23 mm), the mean clinical activity score was 1.31 ± 0.90 (range: 0–4), and the mean muscle index was 0.59 ± 0.08 (range: 0.43–0.72).

**TABLE 1:** Demographic and clinical characteristics of all subjects.

Parameters	Control, *n* = 27	Non-DON, *n* = 32	*P*-value
Eyes	27	32	–
Age, years	41.46 ± 6.72	42.03 ± 9.59	0.792
Sex, female: male	15 : 12	17 : 15	0.852
Spherical equivalent, D	-0.85 ± 1.38	-0.37 ± 1.24	0.178
Intraocular pressure, mmHg	15.27 ± 1.93	16.00 ± 2.62	0.232
Best corrected VA, logMAR	-0.02 ± 0.04	-0.00 ± 0.03	0.538
Mean deviation of VF, dB	-1.27 ± 1.29	-1.07 ± 1.17	0.561
Pattern standard deviation of VF, dB	2.04 ± 0.52	2.14 ± 0.52	0.488
Exophthalmometry, mm	–	19.03 ± 2.44	–
Clinical activity score	NA	1.31 ± 0.90	–
Muscle index	–	0.59 ± 0.08	–

Values for continuous variables are mean ± standard deviation for all subjects in each group.

Control, healthy controls; DON, dysthyroid optic neuropathy; VA, visual acuity; VF, visual field; –, not performed; NA, not applicable.

### Contrast sensitivities to L-, M-, S-cone Gabors

**FIGURE 3:**
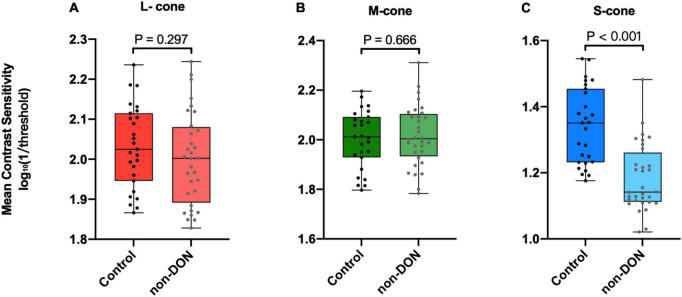
Contrast sensitivities to L- **(A)**, M- **(B)**, and S-cone **(C)** Gabors of the control and non-DON group.

Figure 3 shows the boxplots of mean contrast sensitivity to L-, M-, and S-cone Gabors. To explore whether there was a selective cone-mediated deficit in non-DON patients, we performed an independent sample *t*-test between the non-DON and control groups. We found that differences in L- or M-cone contrast sensitivities between the two groups were not significant (*P*s > 0.29, Figures 3A,B). However, the observers’ sensitivities to S-cone Gabor were significantly different between the two groups (*P* < 0.001, Figure 3C).

### Receiver operating characteristic curve analysis of chromatic vision

**FIGURE 4:**
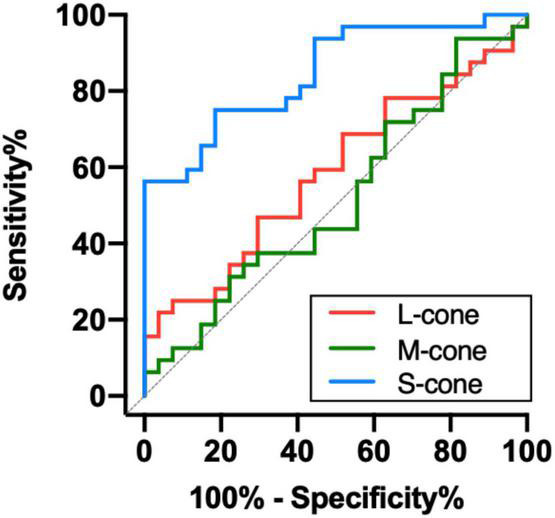
Receiver operating characteristic analysis of the chromatic sensitivity in the TAO patients without clinical signs of DON.

To determine the ability of chromatic sensitivity and to detect the early visual function changes in non-DON patients, we performed the ROC curve analysis. As expected, sensitivity to S-cone stimuli had the highest index to discriminate between non-DON and control eyes (Table 2 and Figure 4).

**TABLE 2:** ROC curve analysis of cone contrast sensitivities in TAO patients without clinical signs of DON.

Contrast sensitivity	AUC	Cut off	Sen, %	Spe, %	*P*
L-cone	0.585	1.98	70.4	46.9	0.263
M-cone	0.483	2.01	55.6	56.2	0.825
S-cone	0.846	1.23	81.5	75.0	<0.001

ROC, receiver operating characteristic; AUC, areas under the ROC curve; Sen, sensitivity; Spe, specificity.

### Associations between S-cone contrast sensitivity and clinical parameters

**FIGURE 5:**
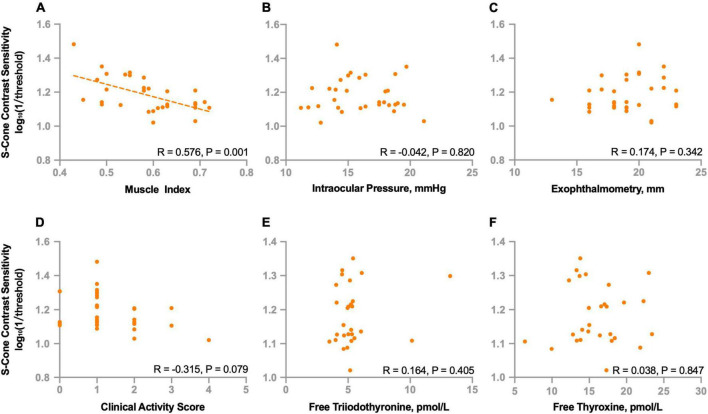
Correlations between S-cone contrast sensitivity and clinical parameters in non-DON patients. Correlations between S-cone contrast sensitivity and muscle index **(A)**, intraocular pressure **(B)**, exophthalmometry **(C)**, clinical activity score **(D)**, free triiodothyronine **(E)** and free thyroxine **(F)**.

To further investigate the potential factors that might be associated with deficits of S-cone in the non-DON group, we evaluated the relationships of S-cone contrast sensitivity and muscle index, intraocular pressure, exophthalmometry, clinical activity score, free triiodothyronine, and free thyroxine. We found the S-cone contrast sensitivity was significantly correlated with the muscle index (*R* = 0.576, *P* = 0.001, Figure 5A) but not with intraocular pressure, exophthalmometry, clinical activity score, free triiodothyronine, or free thyroxine (*P*s > 0.07, Figures 5B–F).

We also investigated the relationships between S-cone contrast sensitivity and other functional indicators. We found no correlations between S-cone contrast sensitivity and best corrected VA (*R* = −0.099, *P* = 0.591), mean deviation (*R* = −0.017, *P* = 0.927) or pattern standard deviation (*R* = 0.102, *P* = 0.578) of VF.

## Discussion

In the current study, we investigated whether there was a selective deficit mediated by S-cone in TAO patients who did not show clinical signs of DON. We show new evidence that the contrast sensitivity to S-cone stimuli is selectively decreased in TAO patients who have normal VA, VF, optic head appearance and no obvious signs of orbital apex crowding. In addition, the degree of S-cone deficits seems to be significantly associated with enlarged extraocular muscles.

TAO is characterized by enlarged extraocular muscles and increased orbital fat, leading to evaluated pressure within the bony cavity. Persistent high orbital tension and the swelling of the extraocular muscles at the orbital apex result in optic nerve dysfunction (Barrett et al., 1988; Giaconi et al., 2002). Despite the advent of several functional tests, such as those that measure VA, VF, pupillary light reflexes and visual evoked potential, that have been used to monitor the changes of optic nerve function of DON, early detection of optic nerve dysfunction has been still a challenge. To illustrate, previous studies showed that 50–70% patients with DON had a best corrected VA of 20/40 or better, and 76% of cases were bilateral with no relative afferent pupillary defect (Dickinson and Perros, 2010). It is necessary for DON patients, especially those with optic nerve dysfunction, to make a prompt recognition and management in order to prevent permanent vision loss.

Previous studies, using Ishihara plates and Hardy-Rand-Rittler plates, show that changes in color vision are an important clinical symptom of DON, and potentially informative when one aims to detect the incidence of DON (Neigel et al., 1988; Mckeag et al., 2007; Wong et al., 2018). Quantitative and sensitive color vision tests are crucial for the detection of optic nerve dysfunction before the development of irreversible visual impairments. The cone contrast test uses the “up-down staircase” method which is designed to estimate the threshold at a fixed performance level in a block of trials to measure cone contrast sensitivity (Cornsweet, 1962). It can also provide both quantitative evaluations of color vision and investigation of the type and severity of color vision deficits. Therefore, we selected the cone contrast test to examine whether there was a selective cone-mediated deficit in TAO patients without clinical signs of DON. Our results provide new evidence that selective S-cone deficits occur even if TAO patients have normal VA, VF, optic nerve appearance and no obvious signs of orbital apex crowding. We infer that deficits of S-cone might be the earliest detectable functional impairment in the progression of DON development.

The ROC curve analysis revealed that the sensitivity to S-cone had a higher discriminating power than the sensitivity to L- and M-cone before the clinical signs of DON occurred. The results are similar to those in the studies of Garip-Kuebler et al. (2020, 2021), which showed that tritan deficiency could be an early sign of DON. Moreover, Tanner et al. (1995) found that chromatic sensitivity, especially in the tritan axis, significantly decreased in DON patients who experienced severe sight loss. Our study, together with these previous reports, suggests that deficits of S-cone might be one of the most reliable indicators of the presence of DON.

There has been accumulating evidence that many other optic nerve relevant diseases, such as multiple sclerosis (Anssari et al., 2019), diabetic retinopathy (Ong et al., 2003), and glaucoma (Drance et al., 1981; Adams et al., 1982; Bayer et al., 2020), also suffer from selective S-cone deficits before the clinical signs appear. The occurrence of selective S-cone deficits in the early stage of optic nerve dysfunction might be related to the unevenly distributed population of cone photoreceptors. It is well known that S-cone photoreceptors are relatively rare in the retina. Even it is known that L-, M-, and S-cones have same sensitivity to pathological harm, a slight damage to S-cones could be more lethal to the physiology due to its sparsity than that to L- and M-cones (Mollon, 1982). Another possible reason for the selective S-cone deficits is that the S-cones themselves might be more fragile than L- and M-cones. The membranes of S-cones are more permeable to toxins, and the connections between S-cones and pigment epithelium are less robust. This might cause S-cones to be more sensitive to subtle damage (Demonasterio et al., 1981).

Mechanical compression is the predominant cause of visual impairments in DON. Thickened extraocular muscles have been found in about 70% of TAO patients and they have been found to be associated with in increasing the risk for DON (Wiersinga et al., 2013). We found a significant correlation between the S-cone contrast sensitivity and the muscle index. This indicates that the deficits of S-cone might be associated with mechanical compression induced by enlarged extraocular muscles. Also, effects of thyroid hormone in the maintenance of cone opsin expression by hypothyroidism have been investigated in human retinal organoids and animal models (Glaschke et al., 2011; Eldred et al., 2018). Additionally, Cakir et al. (2015) found that S-cone deficits still persisted after euthyroidism was achieved in patients with hypothyroidism. Previous studies reported that uncontrolled hypothyroidism might contribute to the development and progression of TAO (Karlsson et al., 1989; Tallstedt et al., 1994; Perros et al., 2005). We speculate that a period of uncontrolled hypothyroidism of TAO patients might also contribute to the involvement of S-cones although there was no correlation between S-cone contrast sensitivity and thyroid hormone levels. We plan to conduct longitudinal follow-up studies to evaluate the relationship between S-cone deficits and dynamical changes of thyroid hormone.

There were several limitations in the current study. First, to draw more reliable conclusions about the selective deficits of S-cone in TAO patients without clinical signs of DON, a larger sample size of subjects would be expected in future studies. Second, since the current study mainly focused on the early visual impairments of TAO, we excluded the patients with reduced VA, VF defects, relative afferent pupillary defect, optic disk edema and evident orbital apex crowding. We will conduct further studies to investigate the cone contrast sensitivity from mild to severe conditions. Moreover, due to the cross-sectional, non-interventional design of the study, we cannot determine whether the S-cone function changes in non-DON patients found in the current study increase the risk of DON development. A longitudinal study is required to follow TAO patients, especially those with decreased S-cone contrast sensitivity. Finally, we did not evaluate retinal structure in our study. Further investigations in the correlations between cone contrast sensitivity and retinal structure are needed to better understand the color vision impairment in TAO.

Since our study does not include patients with clinical signs of DON or longitudinal follow-up of these patients, it is unclear whether S-cone contrast sensitivity could also serve as a sensitive functional vision predictor for DON development. However, our study, along with previous reports on color vision in DON (Tanner et al., 1995; Garip-Kuebler et al., 2020; Garip-Kuebler et al., 2021), suggests that sensitivity to S-cone could serve as an early indicator of visual impairments that is correlated with DON in TAO patients. Evaluating the sensitivity to S-cone in TAO patients before the occurrence of clinical signs of DON may help clinicians in performing a quick diagnosis and an early intervention in the early stage of DON.

## Data availability statement

The raw data supporting the conclusions of this article will be made available by the authors, without undue reservation.

## Ethics statement

The studies involving human participants were reviewed and approved by the Ethics Committee of the Eye Hospital of Wenzhou Medical University. The patients/participants provided their written informed consent to participate in this study.

## Author contributions

YT and WW conceived and designed the study and provided critical revision to the manuscript. HJ and XY conducted the study and wrote the initial draft of the manuscript. HJ, XY, SC, MW, XH, JY, WL, and MX analyzed and interpreted the data. All authors contributed to read and approved the submitted version.

## Footnotes

1
www.sensingm.com

2
https://www.datacolor.com
